# Oral administration with a traditional fermented multi-fruit beverage modulates non-specific and antigen-specific immune responses in BALB/c mice

**DOI:** 10.1371/journal.pone.0233047

**Published:** 2020-05-11

**Authors:** Jamie Bernadette A. Sy, Tsui-Chun Hsu, Aniket Limaye, Je-Ruei Liu

**Affiliations:** 1 Institute of Biotechnology, National Taiwan University, Taipei, Taiwan; 2 Department of Physical Sciences and Mathematics, College of Arts and Sciences, University of the Philippines Manila, Manila, Philippines; 3 Department of Animal Science and Technology, National Taiwan University, Taipei, Taiwan; 4 Agricultural Biotechnology Research Center, Academia Sinica, Taipei, Taiwan; University of Hawai'i at Manoa College of Tropical Agriculture and Human Resources, UNITED STATES

## Abstract

Fruits have been widely considered as the default “health foods” because they contain numerous vitamins and minerals needed to sustain human health. Fermentation strategies have been utilized to enhance the nutritive and flavor features of healthy and readily consumable fruit products while extending their shelf lives. A traditional fermented multi-fruit beverage was made from five fruits including kiwi, guava, papaya, pineapple, and grape fermented by *Saccharomyces cerevisiae* along with lactic acid bacteria and acetic acid bacteria. The immunomodulatory properties of the fermented multi-fruit beverage, in vivo nonspecific and ovalbumin (OVA)-specific immune response experiments using female BALB/c mice were performed. Administration of the fermented multi-fruit beverage reduced the calorie intake, thus resulting in a less weight gain in mice compared to the water (placebo)-fed mice. In the nonspecific immune study model, the fermented multi-fruit beverage enhanced phagocytosis and T cell proliferation but did not affect B cell proliferation and immunoglobulin G (IgG) production. Analysis of cytokine secretion profile also revealed that the fermented multi-fruit beverage enhanced proinflammatory cytokines interleukin (IL)-6, tumor necrosis factor (TNF)-α, and T helper (Th)1-related cytokine interferon (IFN)-γ production, thus creating an immunostimulatory effect. Nonetheless, in the specific immune study model, the results showed that the fermented multi-fruit beverage decreased the production of proinflammatory cytokines IL-6 and TNF-α production in OVA-immunized mice. Moreover, it also caused a decrease in the production of anti-OVA IgG1, which was accompanied by a decrease in Th2-related cytokines IL-4 and IL-5 production and an increase in Th1-related cytokine IFN-γ production, indicating that it may have the potential to shift the immune system from the allergen‐specific Th2 responses toward Th1-type responses. The results indicate that fermented multi-fruit beverage has the potential to modulate immune responses both in a nonspecific and specific manners.

## Introduction

Fruits have an abundant concentration of vitamin C, dietary fiber, and phytochemicals [1]. Vitamin C acts as a powerful antioxidant that can eliminate free radicals, which cause inflammation or promote cancer [2–4]. It also helps to improve the body’s immunity against harmful pathogens [5,6]. Dietary fiber helps control blood pressure, obesity, cholesterol, and blood glucose [7]. Phytochemicals, such as lycopene, have an anti-inflammatory effect that prevents the growth of cancer cells [8–10]. Although fruits are nutritious, the main problem with fruits is that they are perishable such that it is challenging to meet the global demand for fruit because of the increasing worldwide population. One effective way to solve this problem is through the process of fermentation. Fermentation is a very effective food preservation method from the ancient times [11]. Fermentation of fruits not only increases their shelf life, but also enhances their nutritive value, improves their sensory or flavor features, and reduces their toxicity [12]. Fermented food products are also potential sources of probiotic and biogenic products that can also serve as health supplements for people. Furthermore, since most consumers tend to prefer foods that are ready to eat or ready to drink yet are highly nutritious and health-promoting [13], fermentation of food such as fruits is an excellent way to prepare healthy yet readily consumable food products.

Fermented multi-fruit beverages are commonly made at home in Taiwan. Generally, they are made from the fermentation of selected fruits based on the Five Elements Theory (or the Five Phases Theory). According to the Five Elements Theory, all natural phenomena can be divided into five main groups: wood, fire, earth, metal, and water [14–16]. Each of these five main groups can also contain a limitless number of subcategories such as seasons, emotions, sounds, tastes, colors, internal organs, and more. For instance, according to the order of wood, fire, earth, metal, and water, the corresponding five internal organs are the liver, heart, spleen, lungs, and kidneys, respectively [16–18]. The five hollow organs are the gallbladder, small intestine, stomach, large intestine, and urinary bladder [18]. The main five colors are green, red, yellow, golden, and blue; and the five flavors are sour, bitter, sweet, spicy, and salty. In Chinese traditional medicine, the Five Elements Theory plays an important role when making a diagnosis. For instance, an herb that is colored cyan and has a sour taste can be used to treat disorders affecting the liver and modify its function because they are all in the same group of elements [19]. Furthermore, the Five Elements Theory has a significant influence on the diets of Asian people. In Taiwan, a traditional multi-fruit beverage is made from five different color fruits, including kiwi (green), guava (red), papaya (yellow), pineapple (golden), and grape (blue). These fruits have been shown by numerous studies to have various health benefits. For instance, kiwifruit supports immune function and reduces the likelihood of developing cold- or flu-like illnesses [20]. Papaya has anti-inflammatory and immunomodulatory properties and has been shown to have protective effects against breast cancer and prostate cancer [21–25]. Pineapple contains an enzyme called bromelain, which has been demonstrated to have anti-inflammatory, antithrombotic, fibrinolytic, anticancer, and immunomodulatory effects, in addition to being a wound healing and circulatory improvement agent [26,27]. Pineapple has also been found to help prevent colorectal cancer [28]. Guava can help reduce the risk of prostate cancer and inhibit the growth of breast cancer cells because it is rich vitamin C and lycopene [29–31]. Lastly, the phytochemicals in grapes such as proanthocyanins, anthocyanins, flavanols, resveratrol, and phenolic acids have all been demonstrated to have various health-promoting benefits [32–37]. Various studies have also demonstrated the health-promoting effects of fermented fruits. Fermented papaya was found to exhibit neuroprotective effects in astrocytes through its anti-oxidative properties [38]. In vivo assay results also showed that fermented papaya could help alleviate the progression of hepatocellular carcinoma [39] and could increase the free radical scavenging ability of superoxide dismutase [40]. Lactic acid fermentation was also able to improve the antioxidant capacity of pineapple juice [41]. In vivo experiments showed that fermented grape marc was able to suppress type I allergy in BALB/c mice [42]. Furthermore, a traditional fermented grape-based drink known as hardaliye was also found to increase the antioxidant status of study subjects in a randomized controlled clinical trial [43].

Studies have demonstrated that each of these fermented fruits contains bioactive compounds that are responsible for their health-promoting effects. However, when these fruits are consumed together, the total health-promoting effect of the resulting mixture may be modified due to the interactions among the different components [44]. Modulating the immune system is considered significant for maintaining an excellent overall health status in an individual. The immune system is a highly complex and interactive network of cells, tissues, and organs that create a protective system within the human body. For this reason, the main objective of this study was to investigate the nonspecific immune response and OVA-specific immune response of a fermented multi-fruit beverage.

## Materials and methods

### Microbial strains and culture conditions

All the microorganisms were purchased from the Bioresource Collection and Research Center (BCRC; Hsinchu, Taiwan) and cultured according to the instructions provided by BCRC. The microorganisms used for the preparation of fermented multi-fruit beverage included *Saccharomyces cerevisiae* BCRC 21447, *Lactobacillus acidophilus* BCRC 10695, *Pediococcus dextrinicus* BCRC 12842, *Lactobacillus plantarum* BCRC 10069, and *Acetobacter pasteurianus* BCRC 14145. *S*. *cerevisiae* BCRC 21447 was cultured in yeast extract-peptone-dextrose (YPD) broth (Difco Laboratories, Detroit, MI, USA) at 24°C for 16 h on an orbital shaker at 250 rpm; *L*. *acidophilus* BCRC 10695, *P*. *dextrinicus* BCRC 12842, and *L*. *plantarum* BCRC 10069 were cultured in de Mann, Rogosa, Sharpe (MRS) broth (Oxoid, Basingstoke, UK) at 37°C for 16 h without shaking; *A*. *pasteurianus* BCRC 14145 was cultured in mannitol broth (HiMedia, West Chester, Pennsylvania, USA) at 30°C for 16 h on an orbital shaker at 250 rpm. Before use as the starters for fruit fermentation, 16 h-cultures of *S*. *cerevisiae*, *L*. *acidophilus*, *P*. *dextrinicus*, *L*. *plantarum*, and *A*. *pasteurianus* were diluted in sterile saline to yield a final microbial concentration of 10^6^ colony-forming units (CFU) mL^-1^. The microbial concentrations were measured by traditional colony counting methods.

### Preparation of fermented multi-fruit beverage

The fermented multi-fruit beverage was prepared as described by Xu [45]. Briefly, kiwis, guavas, papayas, pineapples, and grapes were obtained from a local market in Taipei, Taiwan. Each fruit was washed well with water, manually peeled, sliced, shredded in a blender, and filtered through cheesecloth to remove the larger pulp fragments. The blended kiwi, papaya, pineapple, guava, and grape juices were mixed in a ratio of 5:15:70:5:5 (w/w), inoculated with 5% (w/v) of *S*. *cerevisiae*, and incubated at 30°C until the mixture reached a pH of 3.5 (about 5 to 7 days). Then, the yeast-fermented fruit pulp was inoculated with 0.5% (w/v) of *L*. *acidophilus*, 1.0% (w/v) of *P*. *dextrinicus*, 1.0% (w/v) of *L*. *plantarum*, and 2.5% (w/v) of *A*. *pasteurianus*. After incubation at 30°C until reaching a pH of 3.0 (about 14 days), the fermented fruit pulp was vacuum filtered through a filter paper (3-μm pore size) and incubated at 30°C for another 8 weeks. Finally, the fermented fruit pulp was vacuum filtered through a filter paper (0.45-μm pore size), and the resulting fermented multi-fruit beverage was stored at 4°C until used in the proximate analysis and animal experiments.

### Proximate analysis of the fermented multi-fruit beverage

The calorie, protein, total fat, saturated fat, trans fat, carbohydrate, sugar, dietary fiber, sodium, ash, and moisture contents of the fermented multi-fruit beverage were determined according to the standardized methods proposed by the Official Methods of Analysis of AOAC International [46].

### Animal care and treatments

Female BALB/c mice were purchased from BioLASCO (Taipei City, Taiwan) at 6 weeks of age and allowed to acclimatize for 2 weeks before the start of experiments. They were kept on a 12-h light/dark cycle in the Animal Room of the Institute of Biotechnology at National Taiwan University and received a standard diet of rodent chow (Laboratory Rodent Diet 5058; PMI Nutrition International, St Louis, MO, USA) and sterilized tap water ad libitum. The animal room was maintained at 25–29°C and relative humidity of 50–70%. The animal studies and protocols were approved by the Institutional Animal Care and Use Committee of National Taiwan University (NTU105-EL-00101) and was performed in accordance with the Guide for the Care and Use of Laboratory Animals prepared by the Institutional Animal Care and Use Committee (IACUC), National Taiwan University.

For the nonspecific immune tests, the mice were randomly divided into four groups, with 10 animals in each group. The first group served as the control group and received sterile distilled water by daily oral gavage. The other three groups served as treatment groups and received a low dose (10 mL per kg body weight per day), medium dose (20 mL per kg body weight per day), or high dose (40 mL per kg body weight per day) of fermented multi-fruit beverage by daily oral gavage for 6 weeks. During the experiments, food and water were provided *ad libitum*, and body weight and feed intake were measured weekly. At the end of experiment, blood was collected directly from the left ventricle of mouse heart under deep anesthesia with isoflurane inhalation. Then the mice were sacrificed by cervical dislocation and the organs (spleen, lungs, heart, liver, and kidneys) were harvested for further analysis.

For the antigen-specific immune tests, the mice were randomly divided into four groups of 10 mice each. The first group served as the control group and received sterile distilled water by daily oral gavage. The other three groups served as treatment groups and received a low dose (10 mL per kg body weight per day), medium dose (20 mL per kg body weight per day), or high dose (40 mL per kg body weight per day) of fermented multi-fruit beverage by daily oral gavage for 10 weeks. After 4 weeks of feeding, mice were then intraperitoneally injected three times with ovalbumin (OVA; Sigma-Aldrich, St. Louis, MO, USA) in 2 week-intervals. The OVA dosage used for each mouse for the three injections were: 2 μg OVA with complete Freund’s adjuvant for the first injection and 10 μg OVA with incomplete Freund’s adjuvant for the second and third injections. One week after each OVA injection, retro-orbital blood was collected from each mouse under mild isoflurane anaesthesia for analysis of the production of OVA-specific IgG1 and IgG2a antibodies. During the experiment, diets and water were provided *ad libitum*, and body weight and feed intake were measured weekly. Two weeks after the third OVA injection, blood was collected directly from the left ventricle of mouse heart under deep anesthesia with isoflurane inhalation. Then the mice were sacrificed by cervical dislocation and organs (spleen, lung, heart, liver, and kidney) were harvested for further analysis.

### Phagocytosis assay

At the day of the sacrifice, 0.1 mL of blood was put in heparinized tubes, and phagocytic activity was measured using the pHrodo Green E. *coli* BioParticles Phagocytosis Kit (Thermo Fisher Scientific, Inc., Waltham, MA, USA) according to the manufacturer’s protocol. Briefly, whole blood was incubated with and without the *E*. *coli* bioparticles, both at 0°C and 37°C for 15 min. Erythrocytes were then lysed, centrifuged, and washing twice. The final white blood cell pellet was then resuspended in buffer, and phagocytosis was measured by flow cytometric analysis using a Cytomics FC500 Flow Cytometry System (Beckman Coulter, Inc., Miami, FL, USA) with a 488-nm argon laser and a 533-nm emission filter. Percentage of phagocytosis was calculated by dividing the number of fluorescent cells by that of total cells.

### Splenocyte preparation and culture

The mouse spleens were excised and stored in Dulbecco’s phosphate-buffered saline solution (Gibco, Grand Island, NY, USA) containing 3% fetal bovine serum (FBS; Gibco, Grand Island, NY, USA) and 1% antibiotic-antimycotic (Gibco, Grand Island, NY, USA) on ice. The organs were then gently pressed with the head of a syringe plunger and passed through a cell strainer. The resulting cell pellet was treated with 2 mL of ACK lysis buffer (Gibco, Grand Island, NY, USA) for 1 min to eliminate red blood cells, washed twice with Hank’s balanced salt solution (Gibco, Grand Island, NY, USA) with 0.5% antibiotic-antimycotic, and then resuspended with complete Roswell Park Memorial Institute (RPMI)-1640 medium (Gibco, Grand Island, NY, USA) supplemented with 10% FBS and 0.5% antibiotic-antimycotic. Cell count and viability were determined with a hematocytometer using the trypan blue exclusion method.

### Lymphocyte subset immunophenotyping

After harvesting, 4×10^5^ splenocytes were placed in tubes and washed thrice with filter-sterilized phosphate-buffered saline (PBS; 0.1 M, pH 7.4) containing 2% FBS and 0.1% NaN_3_. Cells were then blocked with a CD16/32 antibody (BioLegend, San Diego, CA, USA) and then incubated on ice for 10 min. After blocking, the cells were washed with the flow cytometry staining buffer and then incubated with the appropriate directly labeled antibodies (BioLegend, San Diego, CA, USA) for 30 min. The antibodies used for cell labeling included fluorescein isothiocyanate (FITC)-conjugated anti-mouse CD4 and peridinin chlorophyll protein (PerCP)-conjugated anti-mouse CD8a antibody. After labeling, the cells were washed twice, resuspended in flow staining buffer, and analyzed using flow cytometry.

### Splenocyte proliferation assay

Splenocytes were seeded at a density of 2×10^5^ cells per well at a final volume of 200 μL in 96-well flat-bottom plates and were cultured with complete RPMI medium, complete RPMI medium containing 5 μg/ml of concanavalin A (ConA; Sigma-Aldrich, St. Louis, MO, USA), or complete RPMI medium containing 10 μg/ml of lipopolysaccharide (LPS; Sigma-Aldrich, St. Louis, MO, USA). The plates were incubated at 37°C in 5% CO_2_ for 48 h. Finally, the cell proliferation was assessed using the BrDU cell proliferation ELISA kit (Abcam, Cambridge, MA, USA) according to the manufacturer’s protocol. The results were based on the optical density at the wavelength of 450 nm (OD_450_) and expressed as a stimulation index (SI), which was calculated as follows:

$$SI=\left(Mean\;OD_{450}\;of\;mitogen‐stimulated\;cells–Mean\;OD_{450}\;of\;blank\right)/Mean\;OD_{450}\;of\;non‐stimulated\;cells$$

### Serum IgG measurement

Mouse serum was first collected by centrifuging whole mouse blood at 12,500×*g* for 20 min at 4°C. The IgG level in the serum sample was measured using the Mouse IgG total ELISA Ready-SET-Go! Kit (Affymetrix eBioscience, Vienna, Austria) according to the manufacturer’s instructions. Briefly, 96-well plates were first coated with 100 μL per well of pre-titrated, purified anti-mouse IgG monoclonal capture antibody overnight at 4°C, followed by blocking at room temperature for 2 h with 250 μL per well of blocking solution. Plates were then washed with a washing buffer (PBST; PBS containing 0.05% Tween 20) four times and blotted on paper towels to absorb any residual buffer. After blocking, 100 μL of prediluted standards and samples, as well as 50 μL of the pre-titrated, horseradish peroxidase (HRP)-conjugated anti-mouse IgG polyclonal antibody were added to the wells and incubated for an additional of 3 h at room temperature on an orbital shaker. After incubation, the plates were again washed four times, after which 100 μL of the tetramethylbenzidine (TMB) substrate solution was added to each well. Plates were again incubated for 15 min at room temperature on an orbital shaker. The reaction and color development were stopped by the addition of 100 μL of 1-M H_3_PO_4_ to each well. The plates were then read using a microplate reader (VICTOR3 1420 multilabel counter, PerkinElmer Life and Analytical Sciences, Inc., Waltham, MA, USA) at a wavelength of 450 nm.

### Serum OVA-specific IgG1 measurement

OVA-specific IgG1 levels in the sera of OVA-sensitized mice were measured using the Anti-OVA IgG1 (mouse) ELISA kit (Cayman Chemical, MI, USA) according to the manufacturer’s instructions. Briefly, 100 μL of prediluted standards and samples were added to the appropriate wells and incubated for 2 h at room temperature on an orbital shaker. After 2 h, wells were washed four times with a washing buffer, and then 100 μL of the goat anti-mouse IgG1 HRP detection antibody was added into each well. After incubation for 1 h at room temperature on an orbital shaker, plates were again washed four times, after which 100 μL of the TMB substrate solution was added to each well of the plate. After 30 min of color development, the plates were read using an ELISA plate reader at a wavelength of 450 nm.

### Serum OVA-specific IgG2a measurement

OVA-specific IgG2a levels in the sera of OVA-sensitized mice were measured using the Mouse Anti-Ovalbumin IgG2a ELISA kit (Alpha Diagnostic International, San Antonio, TX, USA) according to the manufacturer’s protocol. First, 100 μL of the prediluted standards, samples, and blank were added to predetermined wells and then incubated at room temperature for 1 h. After incubation, the wells were washed four times, and then 100 μL of diluted anti-mouse IgG2a HRP were added. After incubation for 30 min, the wells were again washed five times, and 100 μL of TMB substrate solution were added. The color was allowed to develop for 15 mins in the dark, after which 100 μL of the stop solution were added to each well to stop the enzymatic reaction. Finally, the absorbance of each well was read at 450 nm.

### Cytokine production measurement

Splenocytes were cultured at a density of 1×10^7^ cells per well at a final volume of 1,000 μL in 48-well flat-bottom plates in complete RPMI 1640 medium. The cells were stimulated with either 5 μg mL^-1^ of ConA, 10 μg mL^-1^ of LPS, or 12.5 μg mL^-1^ of OVA. Cultures were incubated at 37°C in 5% CO_2_ for 48 h, after which the cell supernatants were harvested and the levels of various cytokines, including interleukin (IL)-6, tumor necrosis factor (TNF)-α, IL-2, interferon (IFN)-γ, IL-4, IL-5, and IL-10, were measured using LEGEND MAX ELISA kits (BioLegend Inc., San Diego, CA, USA) according to the manufacturer’s protocol.

### Statistical analysis

The data were analyzed using SPSS version 25 software (IBM SPSS, New York, NY, USA). The general linear-model procedure and Duncan’s multiple range test were used to detect differences between means of the treatments. The results are presented as mean ± standard deviation (SD).

## Results

### Effects of fermented multi-fruit beverage on body weight, daily feed consumption, and organ weight

Table 1 shows the results of the proximate analysis of the fermented multi-fruit beverage. The fermented multi-fruit beverage contains 0.5% proteins, 41.3% carbohydrate, 58.0% moisture, and 0.2% ash. It contained no detectable amount of fat.

**Table 1:** The proximate analysis of the fermented multi-fruits beverage.

Parameters	Unit	Obtained Value
Calories	Kcal 100 mL^-1^	265.4
Protein	g 100 mL^-1^	0.5
Fat	g 100 mL^-1^	Not detected
Saturated Fat	g 100 mL^-1^	Not detected
Trans Fat	g 100 mL^-1^	Not detected
Carbohydrate	g 100 mL^-1^	41.3
Sugar	g 100 mL^-1^	39.6
Dietary Fiber	g 100 mL^-1^	0.5
Sodium	mg 100 mL^-1^	7.9
Moisture	g 100 mL^-1^	58.0
Ash	g 100 mL^-1^	0.2

**Fig 1:**
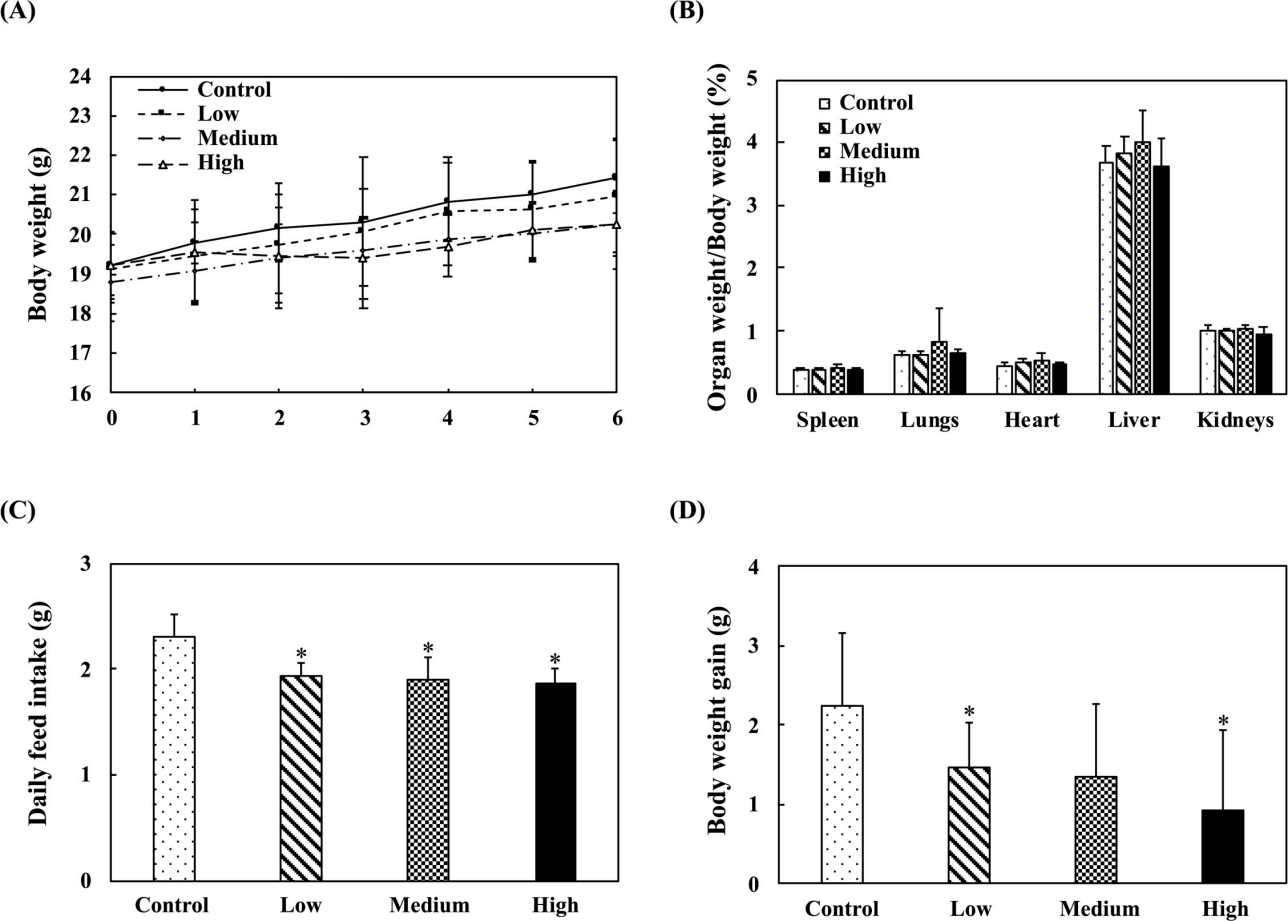
Effect of oral administration of the fermented multi-fruit beverage on body weight, organ weight, daily feed intake, and body weight gain of mice. Control, Low, Medium, and High in the figure indicate control, low dose, medium dose, and high dose groups, respectively. All data are expressed as mean ± SD (*n* = 10). Bars marked with the same letter are not significantly different (*P* > 0.05).

Fig 1 shows the body weight, normalized organ weights, daily feed consumption, and body weight gain of mice in the nonspecific immune study model. Administration of fermented multi-fruit beverage did not affect the body weights and organ weights of the mice (Fig 1A and 1B). However, administration of fermented multi-fruit beverage significantly decreased the daily feed intake of mice, regardless of the dose administered (Fig 1C). In addition, the fermented multi-fruit beverage reduced the amount of body weight gain in mice compared to those in the control group, with the medium or high dose resulting in a significant reduction in body weight gain (Fig 1D).

### Effect of the fermented multi-fruit beverage on CD4/CD8 lymphocyte ratio

**Fig 2:**
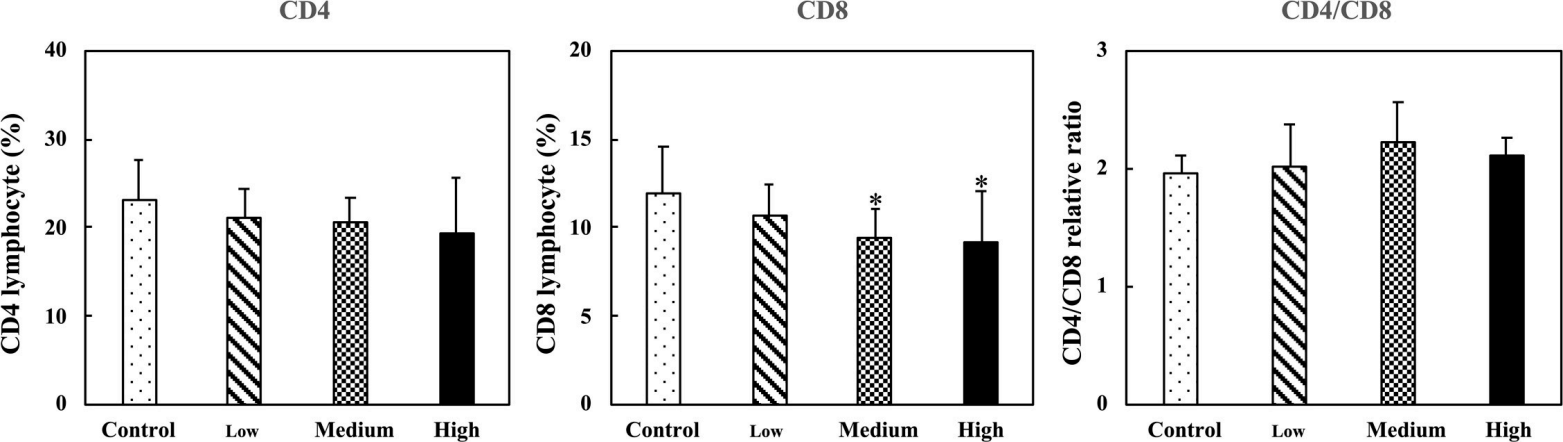
Effect of oral administration of the fermented multi-fruit beverage on the splenetic CD4 and CD8 lymphocyte populations and CD4/CD8 relative ratio of mice. Control, Low, Medium, and High in the figure indicate the control, low-dose, medium-dose, and high-dose groups, respectively. All data are expressed as mean ± SD (*n* = 10). Bars marked with the same letter are not significantly different (*P* > 0.05).

To assess if the fermented multi-fruit beverage had adverse effects on the immune system function, we performed lymphocyte (and subset) immunophenotyping. Oral administration of the medium- and high-dose fermented multi-fruit beverage markedly reduced the CD8 lymphocyte populations. However, the CD4 lymphocyte populations and the CD4/CD8 ratio of mice fed the fermented multi-fruit beverage for 42 days did not differ significantly from that of the controls (Fig 2).

### Effect of the fermented multi-fruit beverage on the proliferative activity of splenocytes

**Fig 3:**
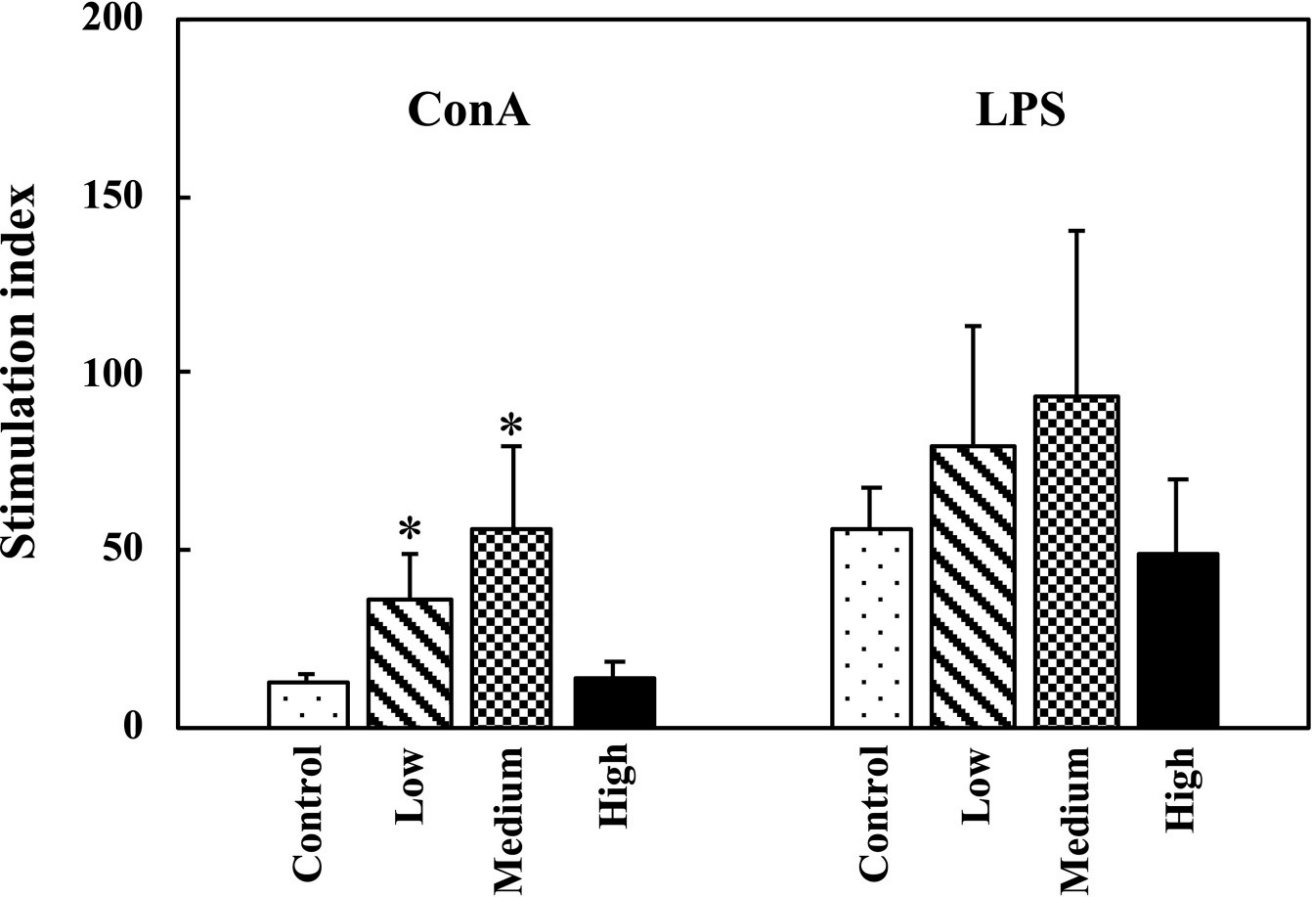
Effect of oral administration of the fermented multi-fruit beverage on the proliferative activity of splenocytes of mice. Splenocytes were stimulated with concanavalin A (ConA) or lipopolysaccharide (LPS) and then their stimulation indexes were determined. Control, Low, Medium, and High in the figure indicate the control, low-dose, medium-dose, and high-dose groups, respectively. All data are expressed as mean ± SD (*n* = 10). Bars marked with the same letter are not significantly different (*P* > 0.05).

Determination of splenocyte proliferation in response to corresponding mitogens is commonly used to evaluate the efficacy of immunomodulatory agents. To assess the effect of the fermented multi-fruit beverage on the cellular immune response, we isolated the splenocytes of mice from the nonspecific immune study model and examined their response to stimulation by ConA and LPS. We found that administration of medium-dose fermented multi-fruit beverages markedly enhanced the proliferation of ConA-stimulated splenic T lymphocytes and LPS-stimulated splenic B cells, although the ConA-stimulated T cell proliferation and LPS-stimulated B cell proliferation did not show a consistent dose-dependent response to the fermented multi-fruit beverage (Fig 3).

### Effect of the fermented multi-fruit beverage on phagocytic activity of peripheral blood lymphocytes

**Fig 4:**
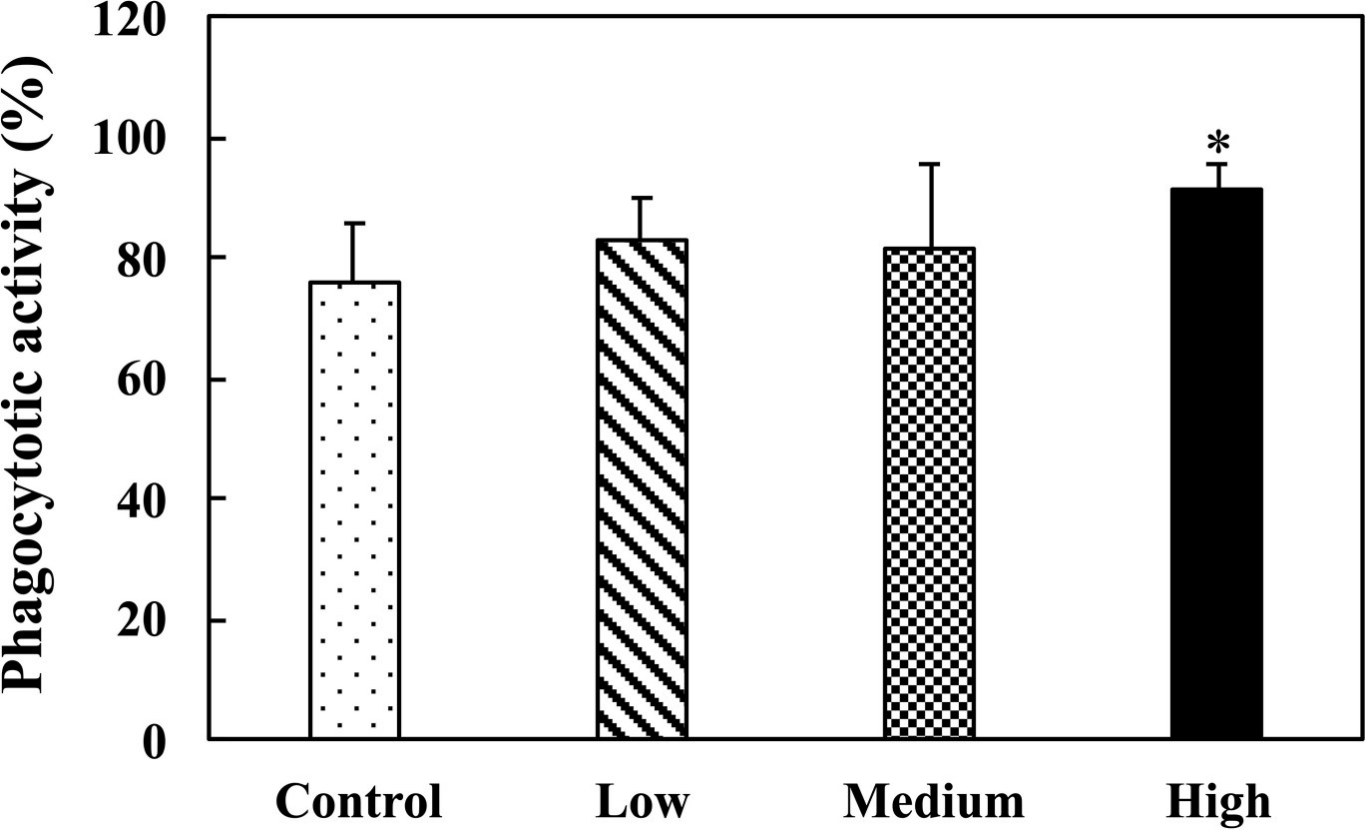
Effect of oral administration of the fermented multi-fruit beverage on the phagocytic activity of peripheral blood lymphocytes of mice. Control, Low, Medium, and High in the figure indicate the control, low-dose, medium-dose, and high-dose groups, respectively. All data are expressed as mean ± SD (*n* = 10). Bars marked with the same letter are not significantly different (*P* > 0.05).

Phagocytosis is considered a crucial component of innate immune defense as it promotes the intracellular destruction of invading pathogens through the action of reactive oxygen radicals and proteolytic enzymes [47]. Fig 4 shows that the high-dose fermented multi-fruit beverage enhanced the phagocytosis of *E*. *coli* by lymphocytes, thus providing evidence for its potential to regulate innate immunity.

### Effect of the fermented multi-fruit beverage on serum antibody secretion

**Fig 5:**
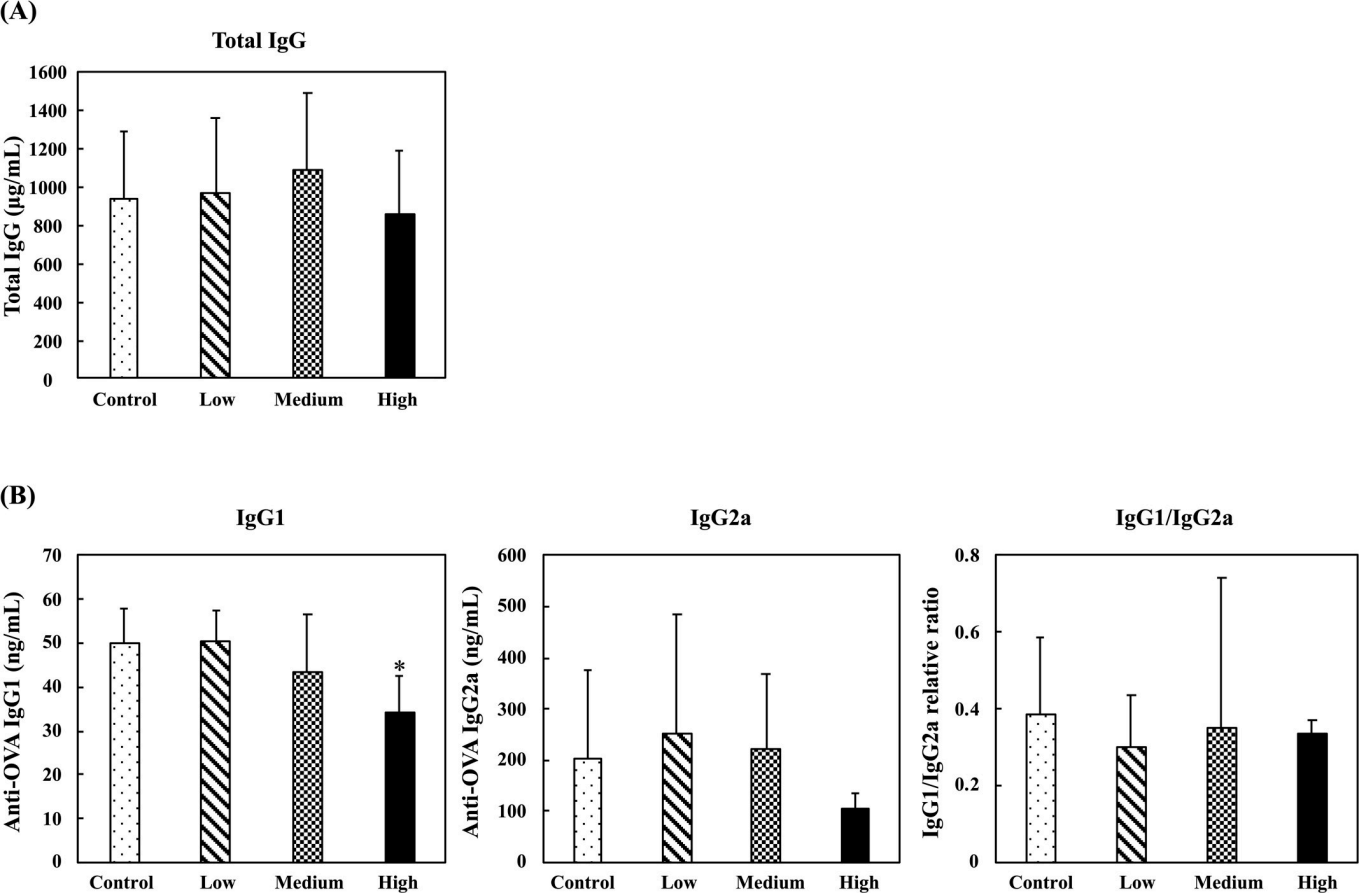
Effect of oral administration of the fermented multi-fruit beverage on serum IgG levels in mice. (A) Serum total IgG levels in mice in nonspecific immune study model. (B) Serum OVA-specific IgG1 levels, OVA-specific IgG2a levels, and OVA-specific IgG1/IgG2a ratio in mice in the OVA-specific immune study model. Control, Low, Medium, and High in the figure indicate the control, low-dose, medium-dose, and high-dose groups, respectively. All data are expressed as mean ± SD (*n* = 10). Bars marked with the same letter are not significantly different (*P* > 0.05).

In the nonspecific immune study model, the effects of oral administration of fermented multi-fruit beverage on serum antibody secretion were evaluated by determining the serum total IgG levels. The results showed that the fermented multi-fruit beverage did not have a significant effect on total serum IgG levels (Fig 5A). In the specific immune study model, the effects of the fermented multi-fruit beverage on serum anti-OVA IgG1 and anti-OVA IgG2a levels were evaluated after mice were immunized thrice with OVA. The results showed that administration of the high-dose fermented multi-fruit beverage significantly decreased the serum anti-OVA IgG1 levels. However, the fermented multi-fruit beverage did not have a significant effect on the serum anti-OVA IgG2a levels or IgG1/IgG2a ratio (Fig 5B).

### Effect of the fermented multi-fruit beverage on cytokine release from splenocytes in the nonspecific immune study model

**Fig 6:**
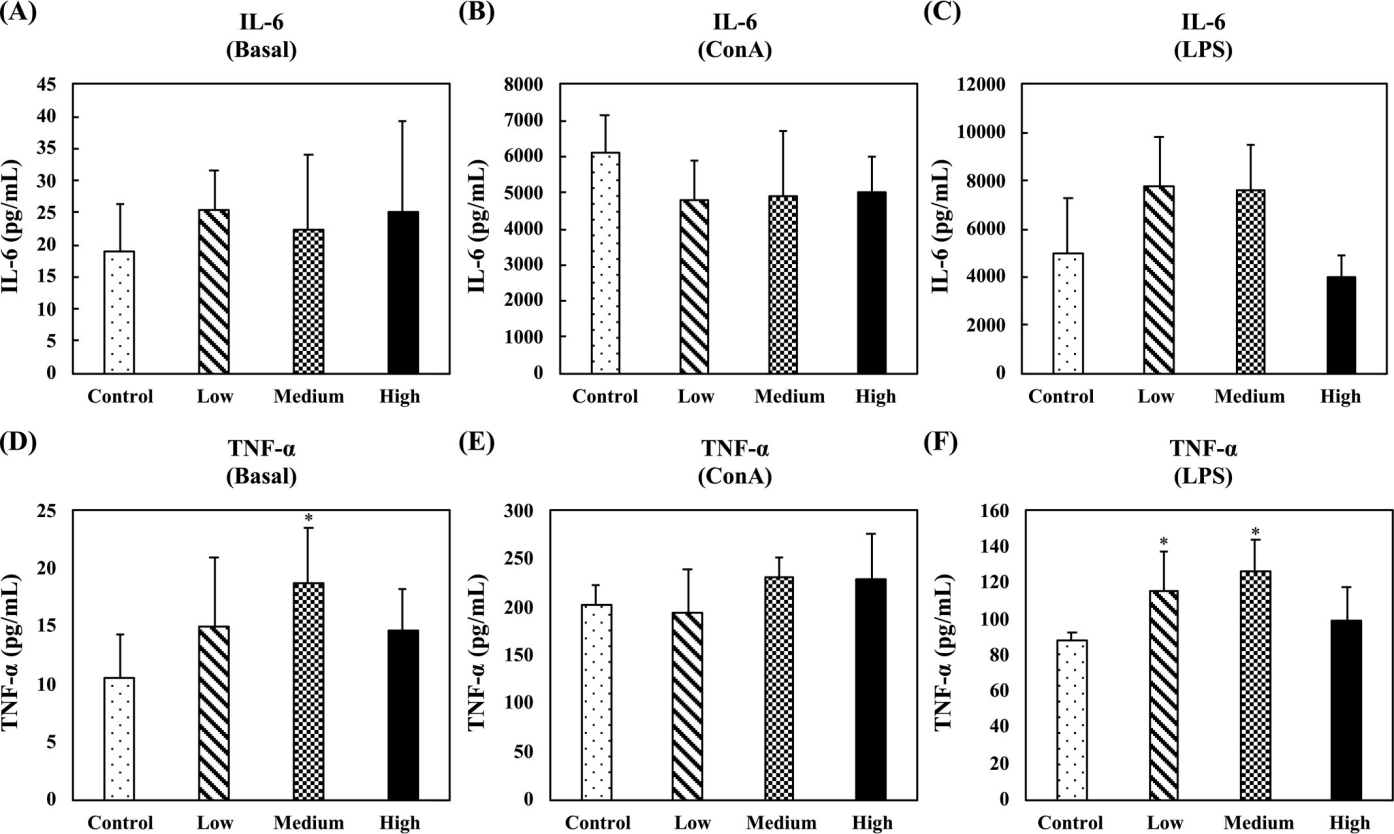
Effect of oral administration of the fermented multi-fruit beverage on proinflammatory cytokines IL-6 and TNF-α released from unstimulated, concanavalin A (ConA)-stimulated, or lipopolysaccharide (LPS)-stimulated splenocytes of mice in the nonspecific immune study model. Control, Low, Medium, and High in the figure indicate the control, low-dose, medium-dose, and high-dose groups, respectively. All data are expressed as mean ± SD (*n* = 10). Bars marked with the same letter are not significantly different (*P* > 0.05).

In the nonspecific immune study model, the effects of oral administration of fermented multi-fruit beverage on cytokine production were evaluated by determining the ex vivo levels of proinflammatory cytokines (IL-6 and TNF-α), Th1-related cytokines (IL-2 and IFN-γ), and Th2-related cytokines (IL-5 and IL-10) in unstimulated, ConA-stimulated, or LPS-stimulated splenocyte culture supernatants. Fig 6 shows the levels of pro‐inflammatory cytokines, IL-6 and TNF-α, in supernatants of unstimulated, ConA-stimulated, or LPS-stimulated splenocytes. In the unstimulated splenocytes, no differences were observed for IL-6 production between any group, and only oral administration of the medium-dose fermented multi-fruit beverage, as compared to the control group, markedly enhanced the TNF-α production (Fig 6A and 6D). In the ConA-stimulated splenocytes, no differences were observed for IL-6 and TNF-α production between any group (Fig 6B and 6E). In the LPS-stimulated splenocytes, oral administration of the low-dose and medium-dose fermented multi-fruit beverages markedly enhanced the IL-6 and TNF-α production (Fig 6C and 6F).

**Fig 7:**
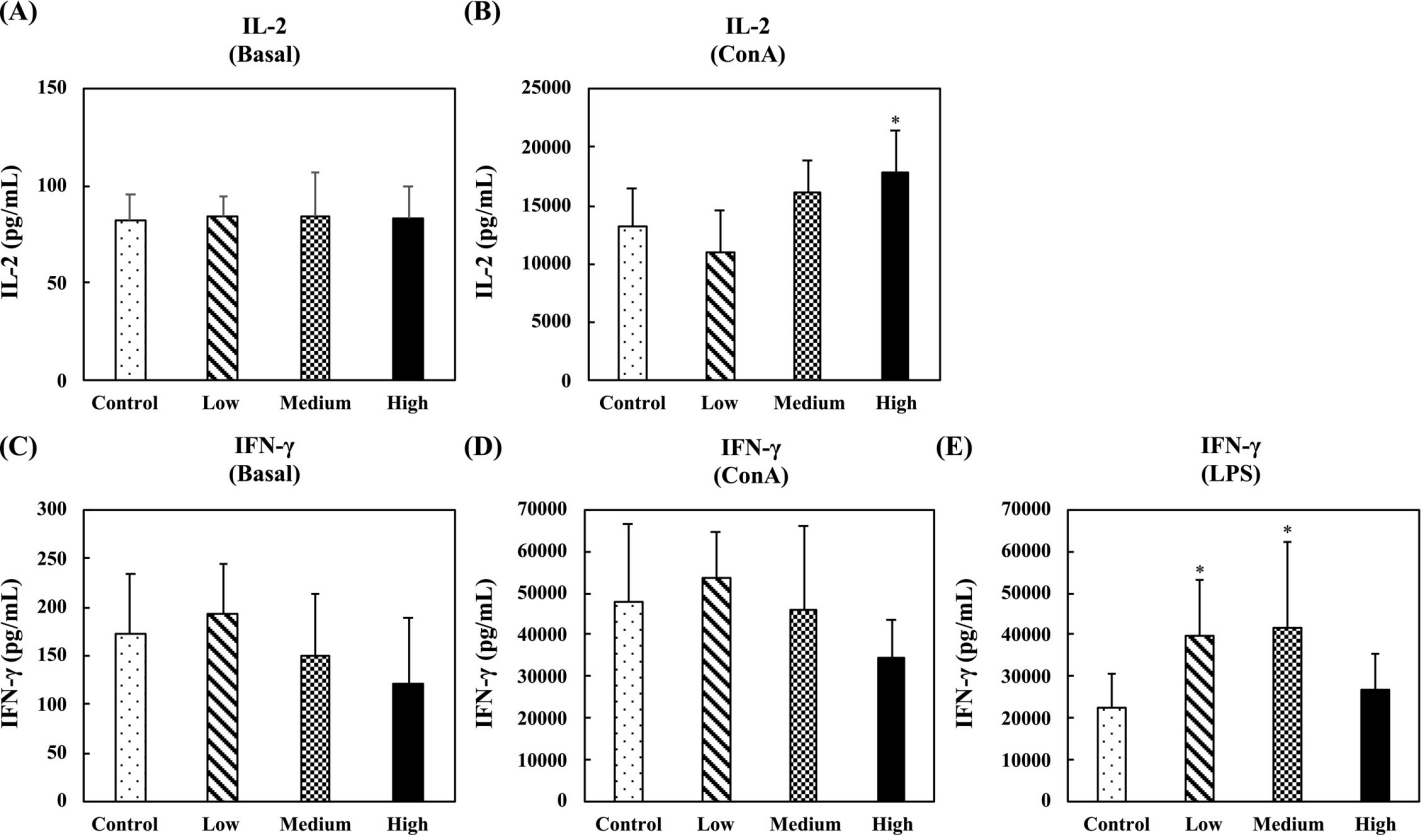
Effect of oral administration of the fermented multi-fruit beverage on Th1-related cytokines IL-2 and IFN-γ released from unstimulated, concanavalin A (ConA)-stimulated, or lipopolysaccharide (LPS)-stimulated splenocytes of mice in the nonspecific immune study model. Control, Low, Medium, and High in the figure indicate the control, low-dose, medium-dose, and high-dose groups, respectively. All data are expressed as mean ± SD (*n* = 10). Bars marked with the same letter are not significantly different (*P* > 0.05).

Fig 7 shows the levels of Th1-related cytokines, IL-2 and IFN-γ, in the supernatants of unstimulated, ConA-stimulated, or LPS-stimulated splenocytes. In the unstimulated splenocytes, no differences were observed for IL-2 and IFN-γ production between any group (Fig 7A and 7C). In the ConA-stimulated splenocytes, oral administration of the high-dose fermented multi-fruit beverage markedly enhanced the IL-2 production as compared to the control group (Fig 7B), and no differences were observed for IFN-γ production between any group (Fig 7D). In the LPS-stimulated splenocytes, the concentrations of IL-2 in the supernatants of splenocytes were too low to detect, and oral administration of the low-dose and medium-dose fermented multi-fruit beverage, as compared to the control group, markedly enhanced the IFN-γ production (Fig 7E).

**Fig 8:**
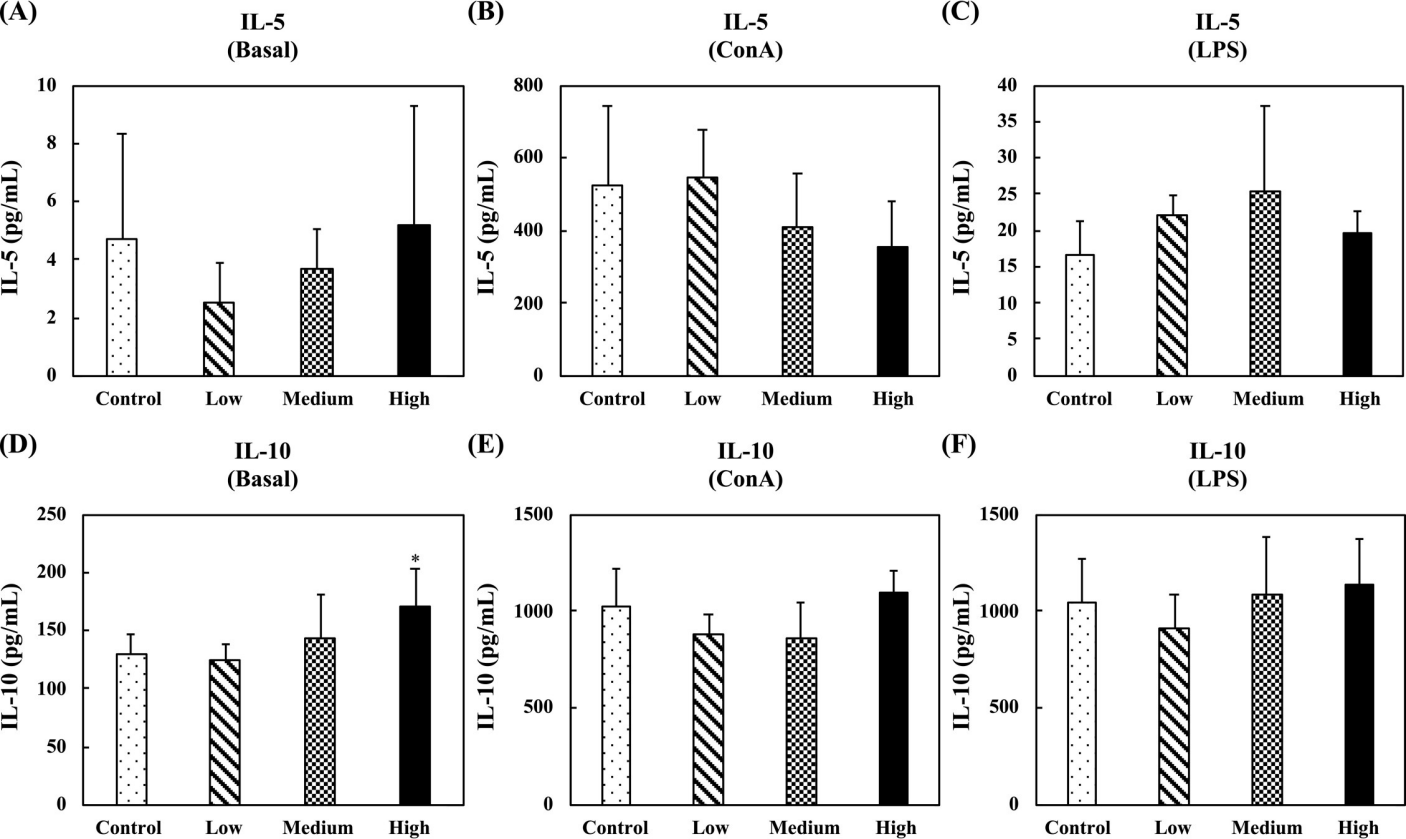
Effect of oral administration of the fermented multi-fruit beverage on Th2-related cytokines IL-5 and IL-10 released from unstimulated, concanavalin A (ConA)-stimulated, or lipopolysaccharide (LPS)-stimulated splenocytes of mice in the nonspecific immune study model. Control, Low, Medium, and High in the figure indicate the control, low-dose, medium-dose, and high-dose groups, respectively. All data are expressed as mean ± SD (*n* = 10). Bars marked with the same letter are not significantly different (*P* > 0.05).

Fig 8 shows the levels of Th2-related cytokines, IL-5 and IL-10, in the supernatants of unstimulated, ConA-stimulated, or LPS-stimulated splenocytes. In the unstimulated splenocytes, no differences were observed for IL-5 production between any group, and only oral administration of the high-dose fermented multi-fruit beverage markedly enhanced the IL-10 production (Fig 8A and 8D). In the ConA- and LPS-stimulated splenocytes, no differences were observed for IL-5 and IL-10 production between any group (Fig 8B, 8D, 8E and 8F).

### Effect of fermented multi-fruit beverage on cytokine release from splenocytes in the OVA-specific immune study model

In the OVA-specific immune study model, the effects of oral administration of the fermented multi-fruit beverage on cytokine production were evaluated by determining the ex vivo levels of proinflammatory cytokines (IL-6 and TNF-α), Th1-related cytokines (IL-2 and IFN-γ), and Th2-related cytokines (IL-4 and IL-5) in the unstimulated, ConA-stimulated, LPS-stimulated, or OVA-stimulated splenocyte culture supernatants.

**Fig 9:**
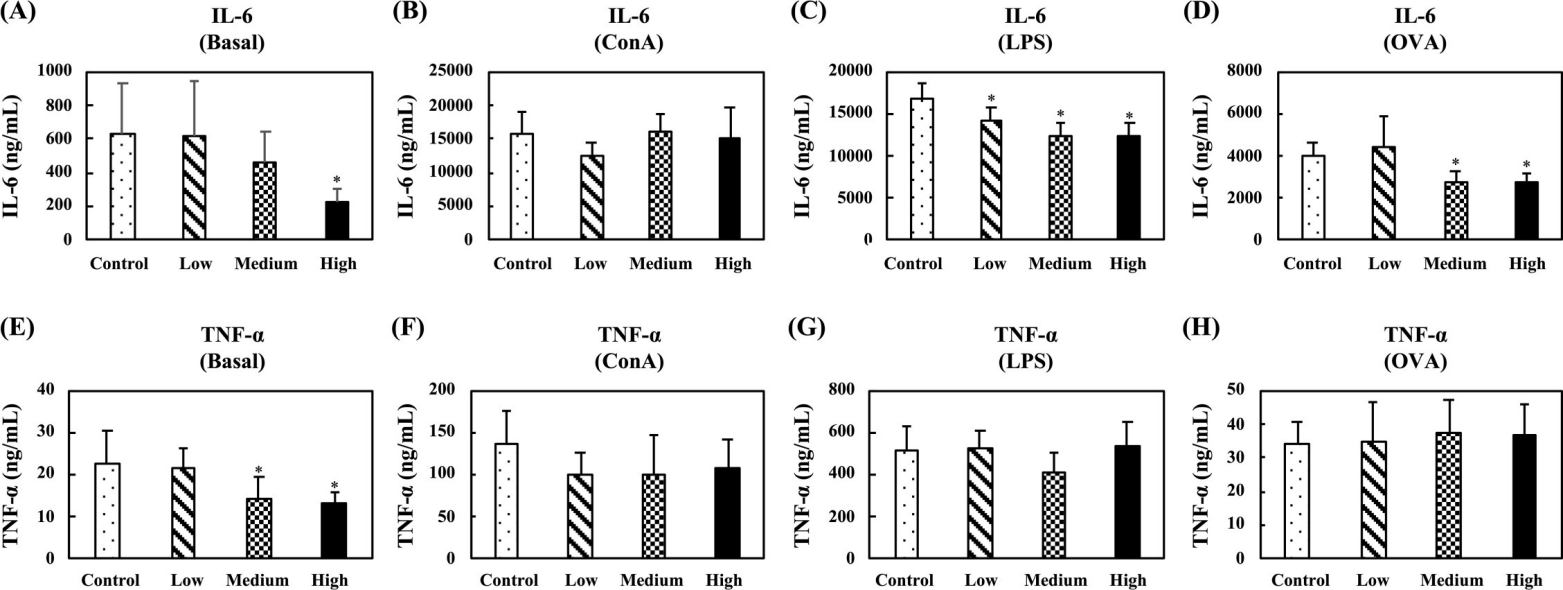
Effect of oral administration of the fermented multi-fruit beverage on proinflammatory cytokines IL-6 and TNF-α released from unstimulated, concanavalin A (ConA)-stimulated, lipopolysaccharide (LPS)-stimulated, or ovalbumin (OVA)-stimulated splenocytes of mice in the OVA-specific immune study model. Control, Low, Medium, and High in the figure indicate the control, low-dose, medium-dose, and high-dose groups, respectively. All data are expressed as mean ± SD (*n* = 10). Bars marked with the same letter are not significantly different (*P* > 0.05).

Fig 9 shows the levels of pro‐inflammatory cytokines, IL-6 and TNF-α, in supernatants of unstimulated, ConA-stimulated, LPS-stimulated, or OVA-stimulated splenocytes. In the unstimulated splenocytes, administration of the high-dose fermented multi-fruit beverage markedly decreased the IL-6 production (Fig 9A), and oral administration of the medium- or high-dose fermented multi-fruit beverage markedly decreased the TNF-α production (Fig 9E). In the ConA-stimulated splenocytes, no differences were observed for IL-6 and TNF-α production between any group (Fig 9B and 9F). In the LPS-stimulated splenocytes, oral administration of the fermented multi-fruit beverage markedly decreased the IL-6 production (Fig 9C) but did not affect the TNF-α production (Fig 9G). In the OVA-stimulated splenocytes, oral administration of the medium- or high-dose fermented multi-fruit beverage markedly decreased the IL-6 production (Fig 9D) but did not affect the TNF-α production (Fig 9H).

**Fig 10:**
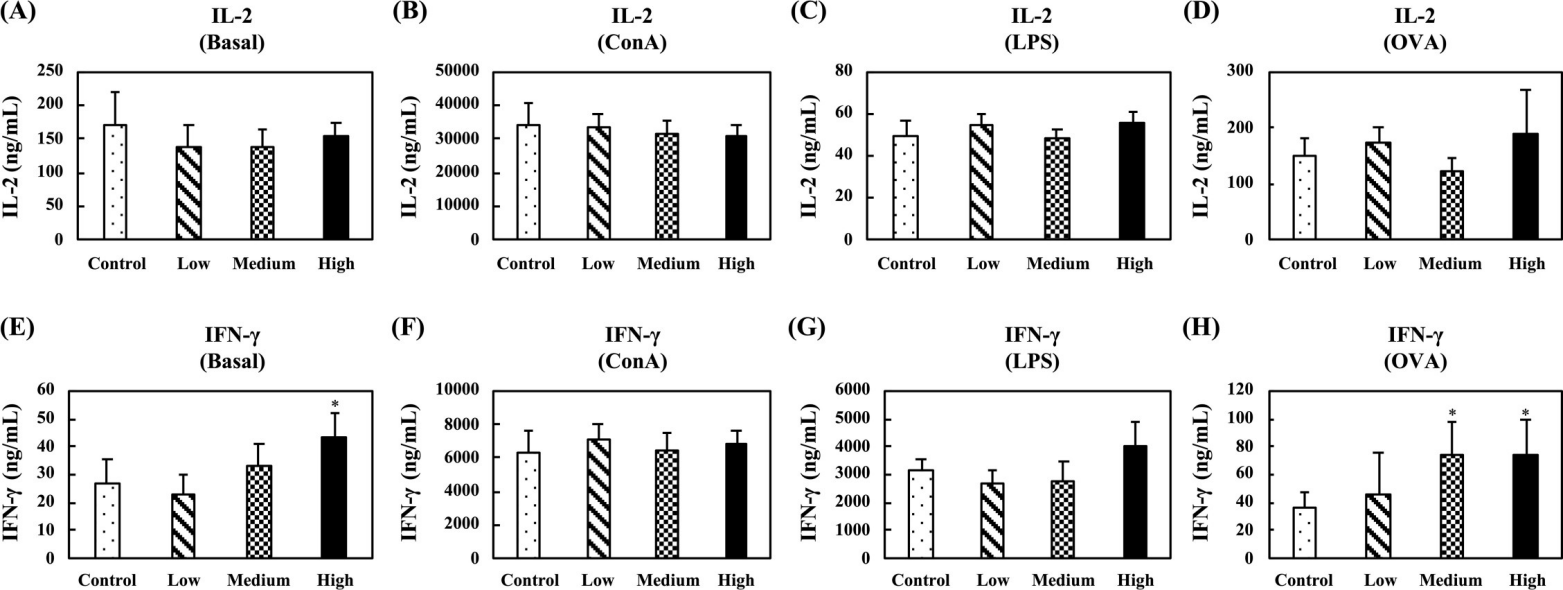
Effect of oral administration of the fermented multi-fruit beverage on Th1-related cytokines IL-2 and IFN-γ released from unstimulated, concanavalin A (ConA)-stimulated, lipopolysaccharide (LPS)-stimulated, or ovalbumin (OVA)-stimulated splenocytes of mice in the OVA-specific immune study model. Control, Low, Medium, and High in the figure indicate the control, low-dose, medium-dose, and high-dose groups, respectively. All data are expressed as mean ± SD (*n* = 10). Bars marked with the same letter are not significantly different (*P* > 0.05).

Fig 10 shows the levels of Th1-related cytokines, IL-2 and IFN-γ, in the supernatants of unstimulated, ConA-stimulated, LPS-stimulated, or OVA-stimulated splenocytes. Oral administration of the high-dose fermented multi-fruit beverage markedly enhanced the IFN-γ production in the unstimulated or LPS-stimulated splenocytes (Fig 10E and 10G); oral administration of the medium- or high-dose fermented multi-fruit beverage markedly enhanced the IFN-γ production in the OVA-stimulated splenocytes (Fig 10H). In the ConA-stimulated splenocytes, no differences were observed for IL-2 and IFN-γ production between any group (Fig 10B and 10F).

**Fig 11:**
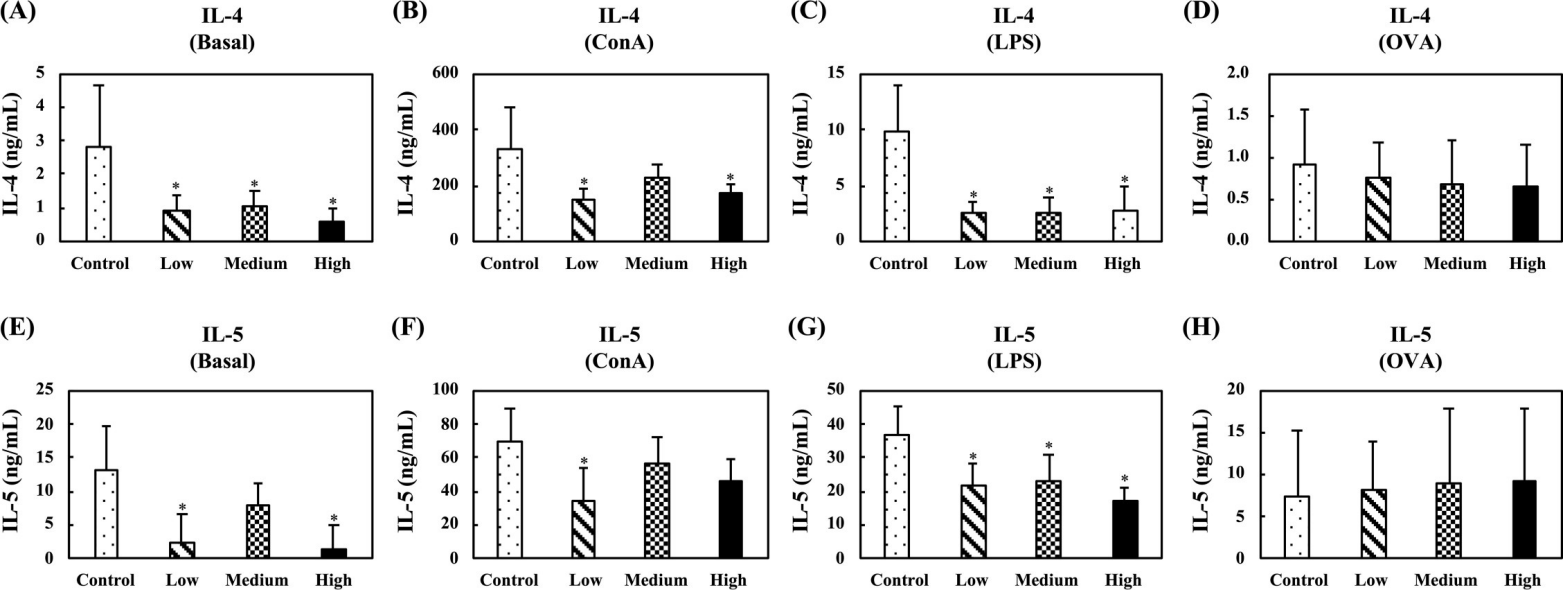
Effect of oral administration of the fermented multi-fruit beverage on Th2-related cytokines IL-4 and IL-5 released from unstimulated, concanavalin A (ConA)-stimulated, lipopolysaccharide (LPS)-stimulated, or ovalbumin (OVA)-stimulated splenocytes of mice in the OVA-specific immune study model. Control, Low, Medium, and High in the figure indicate the control, low-dose, medium-dose, and high-dose groups, respectively. All data are expressed as mean ± SD (*n* = 10). Bars marked with the same letter are not significantly different (*P* > 0.05).

Fig 11 shows the levels of Th2-related cytokines, IL-4 and IL-5, in the supernatants of unstimulated, ConA-stimulated, LPS-stimulated, or OVA-stimulated splenocytes. Oral administration of the fermented multi-fruit beverage markedly decreased the IL-4 production in the unstimulated, ConA-, or LPS-stimulated splenocytes (Fig 11A, 11B and 11C), and oral administration of the low- or high-dose fermented multi-fruit beverages, as compared to the control group, substantially decreased the IL-5 production in the unstimulated, ConA-, or LPS-stimulated splenocytes (Fig 11E, 11F and 11G). In the OVA-stimulated splenocytes, no differences were observed for IL-4 and IL-5 production between any group (Fig 11D and 11H).

## Discussion

Administration of fermented multi-fruit beverage did not affect the body weights. However, administration of fermented multi-fruit beverage significantly decreased the daily feed intake and reduced the amount of body weight gain in mice compared to those in the control group, with the highest dose resulting in a significant reduction in body weight gain (Fig 1). The daily feed intake of the control group and the highest dose group were 2.28 and 1.78 g per mouse per day, respectively. According to the nutritional profile of LabDiet 5058, the gross energy of feed is 4.6 kcal g^-1^. The daily feed caloric difference between the highest dose group and the control group was 2.3 kcal per mouse per day; however, the highest dose group could acquire an extra 0.16 kcal per mouse per day from the fermented multi-fruit beverage (Table 1). Overall, the high-dose group mice still got 2.14 kcal per mouse per day less than those in the control group mice. It might be the primary reason that the weight gain of the high-dose group was lower than that in the control group (Fig 1D). Dietary fiber is often considered an important factor in body weight control; however, it may not be the major reason for the observed effects of the fermented multi-fruit beverage on weight gain control in this study. The control group consumed 0.5 g of feed per mouse per day more than that in the high-dose group; thus, this situation means that the mice in the control group also consumed an extra 0.011 g (2.2% crude fiber in LabDiet 5058 feed) of crude fiber compared to that in the high-dose group. Although the mice in the high-dose group acquired 0.00015 g of dietary fiber from the intake of the fermented multi-fruit beverage (0.5% dietary fiber 100 mL^-1^ of fermented multi-fruit beverage as shown in Table 1), the amount is still significantly less than the amount of dietary fiber consumed by the mice in the control group. We hypothesize that there must be some other component in the fermented multi-fruit beverage that could affect the satiety of mice. The major components of the fermented multi-fruit beverage are carbohydrate and sugar (Table 1), and researches have shown that these two components can prevent weight gain [48], reduce hunger, and increase satiety [49,50]. However, there are still many unknown mechanisms as to how carbohydrates or sugars may affect satiety [48,50]. In our study, the BALB/c mice had access to feed and water *ad libitum*. However, supplementation with the fermented multi-fruit beverage resulted in less feed consumed, and less weight gained compared to the mice in the control group. This result could be attributed to the carbohydrate or sugar contained in the fermented multi-fruit beverage, which might have inhibited the appetite of the mice.

The ratio of T helper cells with the surface marker CD4 to cytotoxic T cells with the surface marker CD8 reflects the immune system health. The CD4/CD8 ratio in the peripheral blood of healthy mice is about 2. A reduced CD4/CD8 ratio is associated with reduced resistance to pathogens. An increased CD4/CD8 ratio is usually observed in the blood of patients with autoimmune diseases [51]. To assess if the fermented multi-fruit beverage had adverse effects on the immune system function, we performed lymphocyte (and subset) immunophenotyping. The CD4/CD8 ratio of mice fed the fermented multi-fruit beverage for 42 days did not differ significantly from that of the controls (Fig 2). Therefore, together with the assessment of the organ weights, particularly the spleen weight (Fig 1B), the fermented multi-fruit beverage was found to have no adverse effects on the immune system function in mice because it did not induce any overstimulation or suppression of the immune system [51].

B cells undergo stimulation and proliferation when they have an encounter with their specific antigens [52]. In the nonspecific immune study model, the fermented multi-fruit beverage did not have a significant effect on total serum IgG levels (Fig 5A). The mice were not exposed to a specific antigen or pathogen; therefore, it is understandable that the fermented multi-fruit beverage did not have a significant effect on stimulation of B cell proliferation, and thus, there is no observed significant increase of total serum IgG levels (Fig 5A). In the specific immune study model, administration of the high-dose fermented multi-fruit beverage significantly decreased the serum anti-OVA IgG1 levels but did not have a significant effect on the serum anti-OVA IgG2a levels or IgG1/IgG2a ratio (Fig 5B). In mice, the production of IgG1 versus IgG2a is widely interpreted as a reflection of differential Th2-Th1 reactivity [53]. Th1 cells promote cellular immune response, including macrophage activation and delayed-type hypersensitivity by secreting IFN-γ and IL-2. In contrast, Th2 cells are generally regarded to be anti-inflammatory and are characterized by cytokine production, including IL-4, IL-5, IL-6, and IL-10. The balance between Th1 and Th2 cell types is considered important for the maintenance of homeostasis. Once the balance between Th1 and Th2 cell types is disturbed, various immunological diseases, such as allergies and inflammation, can occur due to the failure of immune homeostasis. Thus, regulation of the balance between these two types of cells is considered to be essential for immune homeostasis [54]. Several studies have shown that a polarized Th2 response is often observed in patients with allergies [55,56]. Therefore, enhancing a Th1-type immune response is expected to be beneficial for the treatment of allergic disease. In the present study, secretion of OVA-specific IgG1, which is highly dependent on Th2-type cytokines, was markedly inhibited by oral administration of the high-dose of fermented multi-fruit beverage, whereas IgG2a secretion was not inhibited. Therefore, the immune system might be driven toward the Th1-type responses. These results suggest that fermented multi-fruit beverages may be beneficial for the prevention of allergic disorders.

Immunomodulation is defined as changing the physiological immune response, which can be either activated or suppressed using natural or synthetic substances, and the substances responsible for this control are called immunomodulators [57,58]. The immunomodulatory effects of immunomodulators are attributed to the release of cytokines, including ILs, IFNs, transforming growth factor (TGF), TNFs, and others. Cytokines are low molecular weight proteins or peptides secreted by many cell types, particularly immune system cells, that regulate the duration and intensity of the immune response. Cytokines participate in many physiological processes, including the regulation of immune and inflammatory responses [59]. Cytokines are generally divided into the following groups: proinflammatory cytokines (e.g., IL-1, IL-6, and TNF-α), Th1-related cytokines (e.g., IL-2, IL-12, and IFN-γ), and Th2-related cytokines (e.g., IL-4, IL-5, and IL-10) [59]. The immunostimulatory effects of immunomodulators are characterized by the ability to act against infection and allergy through the induction of Th1-related cytokine production. On the other hand, the immunoregulatory effects of immunomodulators are characterized by the ability to decrease allergy, inflammatory bowel disease, autoimmune diseases, and inflammatory responses through the induction of Th2-related cytokine production [57,58]. The effects of cytokines can be analyzed at the protein level in whole blood or peripheral blood mononuclear cells. However, cytokines are rapidly cleared from the circulation and are active at very low concentrations and expressed only following cellular activation [59]. Thus, the analysis of cytokine production in stimulated immune cells is attractive. In this study, we determined the ex vivo levels of proinflammatory cytokines (IL-6 and TNF-α), Th1-related cytokines (IL-2 and IFN-γ), and Th2-related cytokines (IL-5 and IL-10) in unstimulated, LPS-stimulated, or ConA-stimulated splenocyte culture supernatants. We found that exposure of splenocyte to LPS or ConA increased the production of all the cytokines detected in this study (Figs 6–8). These results were consistent with other studies that reported resting immune cells produce a minimal amount of cytokines to meet their basic cellular requirements; thus stimulation is necessary for the measurement of cytokine production [59,60]. Moreover, we found that oral administration of the low-dose and medium-dose fermented multi-fruit beverage markedly enhanced the proinflammatory cytokines, IL-6 and TNF-α, and Th1-related cytokine, IFN-γ, production but did not affect the Th2-related cytokines, IL-5 and IL-10, production in the LPS-stimulated splenocytes (Figs 6–8). Th1-related cytokines, such as IL-2 and IFN-γ, are primarily involved in inflammatory responses by enhancing macrophage-activated intracellular killing of pathogens [61]. During antigen presentation between macrophages and Th1 cells through the major histocompatibility complex (MHC) class 2 molecule, Th1 cells secrete IFN-γ, which is in turn recognized by an IFN-γ receptor on the macrophage cell surface. The IFN-γ then proceeds to activate the macrophage, thereby enhancing its phagocytic activity. In addition, IL-2 is involved with the differentiation of T cells into effector T cells and memory T cells [62]; therefore an increase in IL-2 also correlates well with an increased proliferation of activated T cells. Fig 7 shows that the fermented multi-fruit beverage significantly increased Th1-related cytokines IL-2 and IFN-γ under ConA and LPS stimulation, respectively. According to these data, we suggest that administration of the fermented multi-fruit beverage could increase Th1-related cytokine production (Fig 7) and, in turn, enhance the proliferation of ConA-stimulated splenic T lymphocytes (Fig 3), resulting in an increase of phagocytic activity of peripheral blood lymphocytes (Fig 4). Therefore, these results suggest that the fermented multi-fruit beverage in certain concentrations may have immunostimulatory effects.

OVA is commonly used for studies of the immunogen-specific T- and B-cell-mediated immune responses in hypersensitivity mechanisms [63]. In OVA-sensitized mice, administration of the fermented multi-fruit beverage at certain concentrations decreased Th2-related cytokines (IL-4 and IL-5) production in unstimulated, ConA-stimulated, or LPS-stimulated splenocytes (Fig 11) and increased Th1-related cytokine IFN-γ production in unstimulated, LPS-stimulated, or OVA-stimulated splenocytes (Fig 10). These results correlated well with the results of the OVA-specific IgG1 and IgG2a analyses reported above, in which secretion of OVA-specific IgG1, which is highly dependent on Th2-type cytokines, was markedly inhibited by oral administration of the high-dose fermented multi-fruit beverage, whereas IgG2a secretion was not inhibited (Fig 5B). Several studies have also demonstrated similar results, as observed in this study. For instance, a study investigating the anti-inflammatory effects of a drug called alloferon in OVA-induced asthma in mice has shown decreased levels of IL-5 and IL-6 production, as well as decreased OVA-specific IgG1 but not OVA-specific IgG2a [64]. Another study has also shown that an anti-inflammatory protein kinase inhibitor U0126 decreased the Th2-related cytokines IL-4 and IL-5 and OVA-specific IgG1 production; in addition, OVA-specific IgG2a was not affected [65]. Therefore, we suggest that the administration of fermented multi-fruit beverages could decrease Th2-related cytokine production, and in turn, decreased the secretion of OVA-specific IgG1, resulting in an immune system shift from the allergen‐specific Th2 responses toward the Th1-type responses. Thus, the administration of fermented multi-fruit beverages may be beneficial for the prevention of allergic disorders.

The fermented multi-fruit beverage in this study was made from kiwi, guava, papaya, pineapple, and grape. Numerous previous studies have demonstrated these fruits possess immunomodulatory properties. For example, kiwifruit may enhance immune function [20]; guava and papaya have anti-inflammatory and immunomodulatory properties [21,30]; pineapple has anti-inflammatory effect [27]; grape seed proanthocyanidins could improve functional activation of the immune system [33]. A large number of publications suggested that the immunomodulatory and anti-inflammatory properties of fruits can be attributed to the phytochemicals present in fruits [8–10]. In addition, various studies have also demonstrated the functional activities of fruits can be improved by fermentation [38–43]. Fermentations not only enhance the release of phytochemicals from fruits but also produce large array of new metabolites through microbial bioconversion pathways such as glycosylation, deglycosylation, ring cleavage, methylation, glucuronidation, and sulfate conjugation [66]. In the present study, we found that the fermented multi-fruit beverage may have potential to shift the immune system from the allergen‐specific Th2 responses toward Th1-type responses. However, the detailed molecular mechanism underlying immunomodulatory action as well as the functional constitutes of the fermented multi-fruit beverage are not clear yet and need to be further investigated and identified.

## Conclusions

Our results collectively indicate that the fermented multi-fruit beverage has various health-promoting properties that can be beneficial for humans. In terms of calorie intake, our data suggest that the carbohydrates or sugars in the fermented multi-fruit beverage could play a role in increasing the satiety of mice; thus, the drink could be useful in weight regulation and obesity prevention. In terms of nonspecific immunomodulation, the fermented multi-fruit beverage enhanced phagocytosis and cell-mediated immunity. And lastly, the fermented multi-fruit beverage could also be used to alleviate induced allergies because of its observed immunoregulatory effect in OVA-sensitized mice.
